# Appropriate Use of Proton Pump Inhibitors in General Out Patient Department of a Tertiary Care Center of Kathmandu Valley: An Observational Study

**DOI:** 10.31729/jnma.8913

**Published:** 2025-03-31

**Authors:** Jeetendra Bhandari, Narendra Bhandari

**Affiliations:** 1 Department of General Practice and Emergency Medicine, Patan Hospital, Lagankhel, Lalitpur, Nepal

**Keywords:** *appropriate use*, *general clinic*, *proton pump inhibitor*

## Abstract

**Introduction:**

Proton Pump Inhibitors (PPIs) are widely used medications that suppress gastric acid secretion worldwide. However, they have been linked to an increased risk of chronic kidney disease, hypomagnesemia, and bacterial infections, including C. difficile and acid hypersecretion. This study aimed to identify the appropriateness of PPI prescriptions in general clinics.

**Methods:**

An observational cross-section study was conducted in General out patient department of a tertiary care center of Nepal. Purposive sampling was done. The study included 355 clinical notes from the clinic's out patient department with at least one proton pumb inhibitor prescription. Data was collected, and the proportion of different parameters was calculated.

**Results:**

Appropriate use of proton pumb inhibitor was 255 (57.74%). Among the total study population, 186 (51.22%) were male. The appropriate use of proton pump inhibitor use by faculties was 83 (61.02%) and 68 (33.68%) reported that it was given to prevent Non-steroidal Anti-Inflammatory Drug-related complications. Pantoprazol was prescribed in 256 (74.62%) cases.

**Conclusions:**

This study suggests that PPIs were appropriate more than 50 percent of the time, but a high number of patients have been prescribed PPIs without a clear indication.

## INTRODUCTION

Appropriateness and suitability of healthcare have been discussed since the 1990s.^1^ Antibiotics are the most commonly inappropriately used medicine worldwide, followed by proton pump inhibitors (PPI) which has been in frequent use since 1989 AD.^23^

Studies have shown that PPI has a risk of atherosclerosis, chronic kidney disease, etc, if used longer.^4-6^ Over 110 million PPI prescriptions are filled yearly in the United States.^7^ A study from Austraila suggests that PPI utilization increased by 1318% in one decade.^8^ Similarly, a study from China showed that 50% of PPIs were prescribed appropriately while a study from India showed that only 13.8% of children were prescribed PPIs appropriately.^10,11^

Therefore, this study is designed to determine the appropriate use of PPIs by doctors working in general practice clinic.

## METHODS

This is an observational cross-section study conducted at the General Practice Clinic of the Department of General Practice and Emergency Medicine, Patan Academy of Health Sciences, which is a tertiary care teaching hospital located in Lalitpur, Nepal. The study was conducted between November 2021 and May 2024 AD after obtaining ethical approval from the Institutional Review Committee (Reference number: PMG2203041596).

A purposive sampling method was used, and the maximum sample size calculated was 355. The sample size was calculated as follows.

The sample size was calculated considering 30.7% inappropriate use of PPI taken from a similar study. With a 5% margin of error and 10% non-response rate, the estimated sample size was 355. A purposive sampling was done.^12^

All clinical notes from out patient department (OPD) with at least one PPI prescription were included in the study. Patients who were under the age of fourteen years, clinical notes without symptoms and diagnosis, illegible handwriting and patients already on PPIs before the visit were excluded from the study.

Indications for appropriate PPI prescription in this study were identified following standard guidelines and previous review articles (Table 1).

The principal investigator and a trained colleague collected data weekly at the OPD using photography. which were securely stored, and data was gathered from medical notes using a structured proforma. Consent was obtained form all participants above age of 18 years and assent with participants below 18 years.

Data was entered in EPI-INFO and analyzed in Easy R. Continuous variables were tested for normality. Categorical variables like age, sex, PPI use, etc., were presented as numbers and percentages.

**Figure 1:**
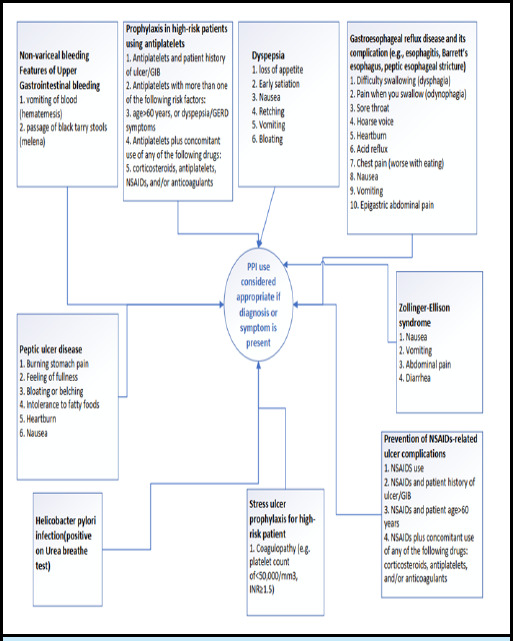
The working definition^13-15^ of appropriate use of PPI.

## RESULTS

The study included 355 patients out of which 186 (51.55%) were male and 114 (32.11%) were in the age group of 40-50 years (Table 1).

The appropriate use of proton pump inhibitor was observed in 255 (57.54%) clinical notes. Clinician of more than 60 years has 39 (81.25%) clinical note where PPIs were prescribed appropriately (Table 1).

**Table 1:** Proportion of appropriate use of Proton Pump inhibitors is based on age group(n=355).

Age Group	Appropriate Use n(%)	Not appropriate Use n(%)
15-20	-	3(100)
20-30	36(50)	36(50)
30-40	66(57.89)	48(42.11)
40-50	37(50)	37(50)
50-60	27(61.36)	17(38.64)
More than 60	39(81.25)	9(10.75)

There were 138 (75.49%) male and 67 (38.95%) female using PPIs appropriately. Amongst the doctors, 83 (61.02%) of faculties used PPIs appropriately (Table 2).

**Table 2:** Proportion of different levels of doctor with (appropriateness of Proton Pump inhibitor use (n=355).

Levels of Doctor	Appropriate Use n(%)	Not appropriate Use n(%)
Medical Officers	43(55.84)	34(44.16)
Resident	79(55.63)	63(44.37)
Faculty	83(61.02)	53(38.98)

**Table 3:** Reasons for appropriate use of Proton Pump inhibitors among patients visiting the general clinic (n=205).

Reasons for the use of Proton pump inhibitors	n (%)
Dyspepsia	6 (2.92)
Features of Upper Gastrointestinal bleeding	18 (8.78)
Gastroesophageal reflux disease and its complication	30 (14.63)
Helicobacter pylori infection (positive Urease breathe test)	18 (8.78)
NSAIDs and patient history of ulcer complication	6 (2.92)
Peptic ulcer Disease	58 (28.29)
Prevention of NSAIDS-related ulcer complication	68 (33.68)

In 258 (73%) clinical notes, diagnosis was included and 63 (33.68%) used to prevent Non-steroidal antiinflammatory drug-related ulcer complications, while 58 (28.29%) were used for peptic ulcer disease (Table 3).

Pantoprazole was used by 265 (74.62%) individuals., (Table 4).

**Table 4:** Frequency and Percentage Distribution of Proton Pump Inhibitors (PPIs) Prescribed Once and Twice Daily (n=355).

PPI Name	Once Daily (n%)	Twice Total (n%)	Daily (n%)
Pantoprazole	212 (75.85)	53 (20)	265 (74.62)
Esomeprazole	37 (88.10)	5 (11.90)	42 (11.83)
Rabeprazole	22 (100)	-	22 (6.21)
Omeprazole	8 (30.77)	18 (69.23)	26 (7.32)

## DISCUSSION

The overall appropriateness of using PPI among the patients visiting the general clinic of the center was 205(57.74%). Different studies have shown different rates of the appropriateness of using PPI. Some studies done in the hospital setting have an appropriateness of use range between 19%-73%, with a mean of appropriate use of 43%.^17-22^ Some of the studies performed in the primary care setting showed the appropriateness of using PPI between 37% and 64%, with a mean of 50%.^12, 23^

A study performed in the community teaching hospital in New Haven County, Connecticut, United States, showed the appropriate use of acid-lowering agents (they used both PPI and H2 blockers as study variables) to be only 35% ( among 226 patients used in the study).^19^ Studies performed in Italy in L. Sacco Hospital, a teaching hospital located in north-west Milan, showed the inappropriate use of acid suppression medication to be 44%(out of 799 patients studied, which used PPI and H2 blockers).^20^ A study that was performed among pulmonary patients in Sweden at Sahlgrenska University Hospital, which is a metropolitan teaching hospital, showed the appropriateness of the use of acid suppression agents to be only 19%(out of 301 patients enrolled; they also used both PPI and H2 blocker as acid suppression agent during the study).^21^ Studies performed in William Beaumont Hospital, Michigan, USA showed appropriateness of the use of acid suppressant(both PPI and H2 blocker) to be 40%(out of 324 patients).^24^

A study performed in Ireland in Midland Regional Hospital at Tullamore, which was a single-day survey of the charts of 157 patients of the hospital, showed the appropriateness of use of PPI to be 67%(out of 48 patients who were prescribed with any one of the PPI).^22^ Studies performed in China, PPI prescriptions for nonhospitalized patients in Beijing, Chengdu, Guangzhou, and Hangzhou, China, over 40 days in 2016 showed the inappropriate use of PPI between 32.6% to 56.8%.^25^ Studies performed in the same center where this study was performed among the admitted patients in surgery and medical ward showed the inappropriate use of intravenous PPI to be as high as 92.3%.^26^

Proton Pump inhibitors are the most used drug worldwide since 1989 AD after the discovery of Omeprazole.^3^ Data have shown that the proportion of PPI users has increased from 0.2% in 1990 to 15.0% in 2014 in the UK. ^27^ Moreover, there are emerging potential adverse effects or risks induced by PPI abuse or in conjunction with other drugs.^4-6, 8^ Why do doctors over-prescribe acid suppression therapy on general medical floors? Practitioners not knowing the correct indications for in-patient anti-secretory therapy may be the leading cause of the misuse of acid suppression.^16^

This study showed that the highest proportion of appropriate use of PPI was in the age group over 60 years. A study on the global burden and demographic profiles of peptic ulcer disease showed that the prevalence of Peptic ulcer disease is higher in the age group above 70 years.^28^ Risk of peptic ulcer complication is 10-fold higher than those over the age of 60 years compared to younger age groups.^16^ The common age group for orthopedics-related degenerative disorders,^29^ cardiovascular-related disorders, is high among the elderly age group.^16^ There is the use of NSAIDs, anticoagulants, antiplatelet therapy, and corticosteroid therapy is high among this age group.^16^ So, there are clearer indications of the use of PPI among these age groups, such as those over 60 years. This can be the reason for the appropriate use of PPI in age groups over 60 years compared to other age groups. In contrary to this, there is also inappropriate use of PPI among the young population, but in comparison to the elderly, it seems to be less.

This study shows that the appropriateness of using PPI is more in males, 75.40% (138 of 183 total males in the study) than in females, 38.95% (67 out of 172 female participants). A study on the global burden and demographic profile of peptic ulcer disease showed that the age and standardized prevalence rate of Peptic ulcer disease is higher among males than females.^28^ But on the contrary, the same study showed higher PUD among females than in males on both spectrums of age spectrums (more than 70 and less than 24 years of age).

This study shows that the most appropriate use of PPI was made by faculties, which was 83(61.02%). A study from the United States suggests that internists there were aware of the 5-6 adverse effects of PPI and said to change the prescribing habit of PPI.^30^

## CONCLUSIONS

This study suggests there was an appropriate use of PPI more than 50 percent of the time, but still, there is a high number of patients who have been prescribed the proton pump inhibitor without a clear indication. This study was done among patients coming to the general clinic.
